# Prevention Trial in the Cherokee Nation: Design of a Randomized Community Trial

**DOI:** 10.1007/s11121-014-0478-y

**Published:** 2014-03-11

**Authors:** Kelli A. Komro, Alexander C. Wagenaar, Misty Boyd, B. J. Boyd, Terrence Kominsky, Dallas Pettigrew, Amy L. Tobler, Sarah D. Lynne-Landsman, Melvin D. Livingston, Bethany Livingston, Mildred M. Maldonado Molina

**Affiliations:** 1 Department of Health Outcomes & Policy, College of Medicine and Institute for Child Health Policy, University of Florida, Gainesville, FL USA; 2 Cherokee Nation Behavioral Health, Tahlequah, OK USA

**Keywords:** Alcohol prevention, Native American, Rural, Community trial, SBIRT, Environment

## Abstract

Despite advances in prevention science and practice in recent decades, the U.S. continues to struggle with significant alcohol-related risks and consequences among youth, especially among vulnerable rural and Native American youth. The Prevention Trial in the Cherokee Nation is a partnership between prevention scientists and Cherokee Nation Behavioral Health to create, implement, and evaluate a new, integrated community-level intervention designed to prevent underage drinking and associated negative consequences among Native American and other youth living in rural high-risk underserved communities. The intervention builds directly on results of multiple previous trials of two conceptually distinct approaches. The first is an updated version of CMCA, an established community environmental change intervention, and the second is CONNECT, our newly developed population-wide intervention based on screening, brief intervention, and referral to treatment (SBIRT) research. CMCA direct-action community organizing is used to engage local citizens to address community norms and practices related to alcohol use and commercial and social access to alcohol among adolescents. The new CONNECT intervention expands traditional SBIRT to be implemented universally within schools. Six key research design elements optimize causal inference and experimental evaluation of intervention effects, including a controlled interrupted time-series design, purposive selection of towns, random assignment to study condition, nested cohorts as well as repeated cross-sectional observations, a factorial design crossing two conceptually distinct interventions, and multiple comparison groups. The purpose of this paper is to describe the strong partnership between prevention scientists and behavioral health leaders within the Cherokee Nation, and the intervention and research design of this new community trial.

## Prevention Trial in the Cherokee Nation: Design of a Randomized Community Trial

The American Academy of Pediatrics recently published a policy statement emphasizing that alcohol use continues to be a major problem among youth, from preadolescence through young adulthood, and additional efforts are needed to prevent and reduce underage drinking (Committee on Substance Abuse 2010). According to the Youth Risk Behavior Survey (Centers for Disease Control and Prevention 2010) conducted within high schools (grades 9–12) across the country, the proportion of youth who reported any use of alcohol or heavy episodic use within the past month was 42 % and 24 %, respectively. Rural youth in general, and rural youth who are an ethnic minority in their community in particular, are at increased risk for alcohol use and getting drunk (Swaim and Stanley 2010). Data from national surveys suggest that Native American high school students have rates of alcohol use similar to the majority White population (Substance Abuse and Mental Health Services Administration 2011). Other studies have documented higher levels of alcohol use (Beauvais et al. 2004) and higher prevalence of past-year alcohol use disorders (National Survey on Drug Use and Health 2007) among Native American youth. Importantly, Native Americans have suffered disproportionately from the negative effects of alcohol (Szlemko et al. 2006). Native Americans are 552 % more likely to die from alcoholism, and significantly more likely to die from unintentional injuries, homicide, and suicide than other Americans (Indian Health Service 2013). Early preventive efforts are urgently needed to reduce these significant health disparities.

Rural communities and Native American populations are both underrepresented populations in clinical and community trial research and give rise to particular challenges for dissemination of evidence-based practices. Challenges to the implementation of evidence-based strategies within rural communities include (a) distance from major metropolitan areas, health centers, and universities; (b) potential limitations on cultural appropriateness, acceptability, and relevance of established strategies; and (c) limited resources of families and communities. To address these challenges, the Prevention Trial in the Cherokee Nation builds on the extant accumulation of evidence by incorporating evidence-based strategies that are inherently adaptable to local culture and incorporating them into an integrated population-level approach for feasible and widespread implementation and optimal reach throughout the community. Further, our partnership incorporates a model of community-based participatory research that also adheres to the strict scientific methods of a controlled trial. This avoids a traditional dichotomy between these two approaches and creates effective working partnerships between academic scientists and their professional peers working in the field. Our goal is creating, implementing, and evaluating a new, integrated population-level intervention designed to prevent underage drinking and associated negative consequences among multicultural youth living in rural high-risk and underserved communities.

## Community-Based Participatory Research Approach

A community-based participatory research (CBPR) approach and selection of evidence-based strategies that are inherently adaptable to individual and community characteristics were envisioned as a way to overcome the three main barriers (distance from major metropolitan areas, health centers, and universities; potential limitations on cultural appropriateness; limited resources of families and communities). CBPR is an orientation to research that engages academic and community partners throughout the research process (Israel et al. 1998; Minkler 2010). Of key principles of community-based research defined by Israel et al. (1998), four are especially relevant in describing the partnership we have developed for the implementation of this trial. These key principles include (a) facilitates collaborative partnerships in all phases of the research, (b) builds on strengths and resources within the community, (c) integrates knowledge and action for mutual benefit of all partners, and (d) promotes a co-learning and empowering process that attends to social inequalities (Israel et al. 1998).

Soon after an NIH request for applications was posted for “multi-component youth/young adult alcohol prevention trials” (RFA-AA-11-001), a team of prevention scientists at the University of Florida (UF) was exploring options for a community partner. They were particularly interested in developing a partnership with a Native American community given the significant alcohol-related health disparities suffered among Native Americans. A mutual colleague introduced the UF team to leaders of Cherokee Nation Behavioral Health and participated in the initial brainstorming meeting.

The Cherokee Nation (CN) is the second largest Native American tribe with nearly 300,000 citizens. About half of the tribal members live within the 14-county jurisdictional service area of the Cherokee Nation in northeastern Oklahoma. It is *not* a reservation. Cherokee citizens comprise a significant proportion of the population within this 14-county region; however these are multi-ethnic rural communities with mostly Native American (10 % to 44 %) and White (44 % to 79 %) populations. Tahlequah, Oklahoma is the capitol of the Cherokee Nation and home to the tri-partite government (i.e., executive, judicial, legislative), which includes a democratically elected principal chief, deputy principal chief and 17-member tribal council. Governmental services include housing, community, education, health and human services, and commerce and career services. The Cherokee Nation operates the largest tribally operated health care system in the U.S.A. Within the health system is Cherokee Nation Behavioral Health, directed by Dr. BJ Boyd, which provides mental health services, substance abuse treatment, and community-based programs promoting mental health. Dr. BJ Boyd, as well as Dr. Misty Boyd, a clinical child psychologist, serve as the CN Co-PIs of the trial. Substance abuse and related consequences are major issues faced by the CN behavioral health team, and they are committed to early prevention and community-based efforts, as well as providing treatment services.

The University of Florida prevention science team has over a 20-year history of conducting community-based prevention trials focused on multiple populations from small, largely White rural communities, to African-American and Hispanic urban populations; from children and adolescents to young adults; and studies of U.S. populations complemented by collaborative global work over the years in Australia, Britain, India, Japan, New Zealand, Norway, Russia, and Tanzania (Komro et al. 2008, 2004; 2001; Wagenaar et al. 2000a). The trials that have included community-level environmental change have incorporated a community-driven organizing approach to lead that change.

During our initial in-person meeting in Tahlequah, it was clear to both teams that this was a mutually beneficial collaboration. We spent an intense and energetic 2 days together learning about each others’ values and passion for child health promotion, as well as productively outlining major plans for the grant proposal, including research and intervention designs. Each team had to commit to the partnership and successful implementation of the trial. For the CN team, it meant committing to additional effort (e.g., community outreach, project supervision, learning new research skills) above and beyond an already hectic schedule. For the UF team, it meant committing to frequent travel, research mentorship, and equality in the partnership. We have structured the project to be co-led by the UF PI (KK) and the CN Co-PIs (MB, BB). However, it is a democratically run project with full participation of all UF and CN team members. We hold multiple weekly conference calls where we debate and make decisions together. In addition to the core project team (UF and CN), we have multiple community partners that also have key roles in specific components of the project including (a) school district superintendents, high school principals, and teachers; (b) Oklahoma Department of Human Services local supervisors and school-based social workers; and (c) local citizens and community organizations.

The Prevention Trial in the Cherokee Nation is a rigorous community trial funded by NIH, using methods of translational science by providing infrastructure, training, and technical assistance to incorporate two evidence-based interventions into rural and underserved communities and schools. The implementation of the trial provides an important example of the development and success of a practitioner–scientist partnership. We have followed key steps in developing practitioner–scientist partnerships as recently outlined (Spoth et al. 2013), including (a) identifying common goals of interest, (b) community leadership development, (c) incorporating role flexibility through shared decision-making, (d) careful measurement of change to produce continuous quality improvement, and (e) equality in partnerships and valuing each others skills and knowledge. The partnership and the integration of the interventions and their evaluation into the established local institutional structures of the Cherokee Nation and involved communities also increases the likelihood of longer-term sustainability following the research trial. The following sections outline the intervention and research design for the trial that have been developed and are being implemented through a strong academic and community partnership.

## Intervention Design

The intervention design builds directly on results of multiple previous experimental research trials with two broad intervention approaches—(a) community environmental change and (b) screening, brief intervention and referral to treatment (SBIRT). Major progress has occurred in science and prevention practice in reducing youth access to alcohol through commercial sources such as bars and stores (Anderson et al. 2009; Wagenaar and Wolfson 1994, 1995). As progress has been made in attenuating commercial access of alcohol to teens (though much remains to be done in most communities), the role of informal social sources is gaining increased attention. Significant challenges remain in understanding how best to reduce availability of alcohol through social sources, particularly from (often slightly older) peers. And, importantly, progress has been less in environments characterized by high alcohol use, other risk factors, and socioeconomic disadvantage (Anderson et al. 2009; Foxcroft et al. 2002; Hawkins et al. 2004; Komro et al. 2004).

On an individual level, there is evidence for short-term reductions in underage alcohol use through SBIRT (Clark et al. 2010; Clark and Moss 2010; Moore and Werch 2009; Wachtel and Staniford 2010; Werch et al. 2010, 2000). The SBIRT approach has also been determined effective with other populations and other risk behaviors, such as heavy alcohol use, body weight, total blood cholesterol, and blood pressure among adults (Kaner et al. 2007; Rubak et al. 2005; Whitlock et al. 2004). The preponderance of previous trials of SBIRT, however, have been focused on single small- to modest-scale institutional settings, such as primary care clinics, hospital emergency departments, or a single or selective set of schools. No trials to date have tested a population-level intervention incorporating features of SBIRT found efficacious in the small-scale or limited institutional settings of previous trials. In short, challenges remain in understanding how to bring SBIRT efforts to scale within communities, broaden population-level relevance and implementation, and sustain preventive effects over time.

The evidence base for these two strategies led to our design of a new community intervention strategy that combines components of strategies at the individual and community level, with innovations designed to strengthen and enhance effects. We selected these two approaches for implementation within our multi-ethnic, rural communities since they are both adaptable and responsive strategies to individual and community differences. A community organizing approach empowers local citizens to create change within their communities based on their values and needs. SBIRT, implemented using motivational interviewing, is designed to be responsive to individual student needs and readiness to change.

We have designed both intervention approaches for *all* high school students in the study communities—a universal, population-level prevention approach. We do *not* single out Native American students or families for the interventions. Available regional and national youth surveys (Centers for Disease Control and Prevention 2010) indicate alcohol use preventive efforts are warranted for youth regardless of their race/ethnicity. Importantly, singling out Native American youth contributes to stigmatization and isolation of Native American youth. Through the partnership between Cherokee Nation Behavioral Health and prevention scientists, as well as epidemiological and formative research, we have selected intervention components that are culturally responsive and relevant, but not culturally limited to one group. The needs for effective prevention and support are high throughout our study communities, and our interventions are carefully designed so no group feels excluded. By updating and combining the most efficacious components from previous research trials on community environmental change, designing a new system for universal and school implementation of an SBIRT intervention, and ensuring cultural relevance, the current trial is designed to advance preventive effectiveness and efficiency in high-need, underserved and multi-ethnic communities.

### Theoretical Framework

**Fig. 1:**
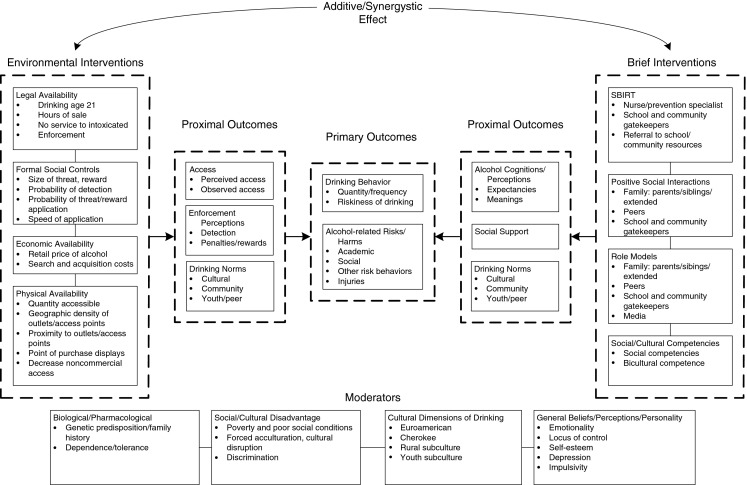
Theoretical framework for the Prevention Trial in the Cherokee Nation

Wagenaar and Perry’s (1994) comprehensive theoretical framework of drinking behavior guided development of our integrated universal preventive intervention, as shown in Fig. 1. We designed a community intervention that builds directly on the original Communities Mobilizing for Change on Alcohol (CMCA) trial and subsequent disseminated model intervention (Wagenaar et al. 2000a; Wagenaar et al. 2000b; Wolfson et al. 2012) with additional evidence-based components. The environmental interventions in the current trial are community-driven and focus on decreasing physical availability and increasing formal social controls on both access to and consumption of alcohol by youth. Intervention features are hypothesized to affect perceived and observed access to alcohol by youth, perceptions of enforcement, drinking norms, drinking behaviors, and alcohol-related risks and outcomes.

We also designed a universal implementation of SBIRT, integrating key components from youth-focused strategies (Clark et al. 2010; Clark and Moss 2010; Moore and Werch 2009; Wachtel and Staniford 2010; Werch et al. 2010, 2000), while expanding relevance and reach with components from effective alcohol and suicide prevention strategies (Isaac et al. 2009; Perry et al. 2000b; Wyman et al. 2008). The expanded SBIRT intervention, CONNECT, will focus on promoting screening with brief intervention using motivational interviewing, positive social interactions and role models, and social and multicultural competencies. The CONNECT intervention is designed to affect alcohol cognitions, expectancies, social support and bonding, drinking models and norms, drinking behaviors and alcohol-related risks, in addition to serving the conventional role of SBIRT in identifying and nudging especially high-risk individuals into more intensive treatment.

### Environmental Intervention: *CMCA*

Direct-action community organizing, documented as effective in multiple previous trials, is used to address community identified issues related to alcohol use and access to alcohol among youth. Action teams were formed in each intervention community, assisted by a community organizer trained in community organizing methods such as those used in D.A.R.E. Plus, Project Northland Chicago, Tobacco Policy Options for Prevention, and CMCA randomized trials (Forster et al. 1998; Komro et al. 2008; Perry et al. 2000a; Wagenaar et al. 2000a, b; Wagenaar and Perry 1994). Goals of the environmental interventions include the following: (a) reduce the number of alcohol outlets that sell to young people; (b) reduce the availability of alcohol to youth from noncommercial sources, such as parents, siblings, older peers, and via kegs and/or at parties; (c) reduce community tolerance of underage drinking and adult provision of alcohol to youth; and (d) ultimately, reduce youth alcohol consumption and alcohol-related problems. The primary, evidence-based intervention, CMCA (Wagenaar et al. 2000a, b; Wagenaar and Perry 1994), has been enhanced by incorporating the latest evidence regarding enforcement checks, hot-spot policing, and the role of earned and paid media.

A local community organizer was hired in each CMCA intervention community. Following local advertisement, we screened the applicant pool and members of the CN team (MB and DP) conducted in-person interviews. Final candidates were interviewed by the UF PI (KK) via phone. We conducted an initial 3-day training led by CN (MB, DP) and UF (KK, AW, SL, BL) team members, as well as a nationally recognized alcohol prevention community organizer (Diane Riibe). A member of the CN team (DP) serves as the Intervention Director and supervises all intervention staff. He, along with the CN Co-PI (MB), hold weekly calls with the organizers and conduct regular site visits. Members of the UF team (KK, AW, SL) participate in the calls every other week. The calls include review and guided feedback based on process documentation completed weekly by the organizers and their oral summary of progress. Formal, in-person booster trainings and strategizing sessions are held in Tahlequah four times each year with members of the CN and UF teams.

A community organizing model was selected as an optimal way to engage a diverse group of local citizens in these multi-cultural towns. Shared values surrounding the protection and promotion of youth well-being and the recognition of alcohol use as a major risk is a motivating factor for many citizens that have committed their time to join their local community action team. Five key principles guide our organizing approach, including (a) empowerment and leadership development of local citizens, (b) reliance on relationship building, (c) mobilization and action of local citizens, (d) community determination of strategies and community ownership, and (e) intentional use of evidence-based strategies for sustainable community change.

We defined six main stages of community organizing and provide guided feedback to the community organizers as they work with their community action teams. The six stages include (a) assessment of community interests through face-to-face, one-on-one or two-on-one meetings with hundreds of community residents; (b) building a base of support through one-on-ones and establishment of a community action team; (c) expanding the base of support through one- or two-on-one meetings, presence and presentations at community events, and media advocacy; (d) development of a plan of action; (e) implementation of actions; and (f) maintenance of effort and institutionalization of change. These six stages are an iterative process, in that they are fluid with movement between and across stages and are meant to be responsive to community needs and capabilities. The UF and CN team provide training and technical assistance in principles of community organizing and on evidence-based prevention strategies. On an ongoing basis, we provide evidence-based fact sheets and action guides that address community needs around reducing underage access to alcohol through commercial and social sources. Action teams meet monthly to discuss community needs, strategize and select appropriate actions, and plan events and actions. All one-on-ones, action team meetings, team actions, and media are documented weekly using standardized forms (described below in the “Implementation Evaluation” subsection).

### SBIRT Intervention: *CONNECT*

The CONNECT intervention includes four key components: (a) twice a year brief one-on-one screening and motivational interviewing sessions delivered to every high school student in the study cohort (class of 2015 and 2016) by a “CONNECT Coach,” (b) training of school staff to reinforce risk identification and communication and support skills, (c) training of community members, and (d) family and community media campaign to reinforce intervention objectives. Goals of CONNECT are to increase bonding, social support and inclusion, and to shift towards more protective alcohol cognitions and norms among high school students and the general population.

Given the challenge of low health care access and utilization among high school students, and the fact that only a small proportion of high school students see a physician or visit a clinic in a given year, we designed the SBIRT intervention to be delivered in schools. We partnered with the Oklahoma Department of Human Services (OKDHS) to provide a full-time school social worker in each CONNECT intervention high school. The CN team along with the local school superintendent and/or principal participated in the interview and hiring process. The school social workers devote 49 % FTE effort to serving as the school’s CONNECT Coach for this trial and the other 51 % FTE they serve as a liaison linking students and their families to relevant community services, which strengthens the partnership between the schools and the OKDHS and enhances sustainability.

The Coaches conduct brief (15 min) one-on-one health consultations in a private school office with each student once each semester (mean number of cohort students per school is 300, range 262 to 369). Coaches communicate with the school principals to work out a mutually agreed upon plan for scheduling sessions. Teachers and students are notified of each scheduled appointment via email or an appointment card. Students are excused from class for their 15-min session. Our implementation of SBIRT is based on NIAAA’s Alcohol Screening and Brief Intervention for Youth: A Practitioner’s Guide guidelines (NIAAA 2011). The brief session includes advice, motivational interviewing, norm-setting messages and referral for follow-up support or specialty treatment, including a brief follow-up session for those referred.

The CN Intervention Director (DP) supervises the project effort by reviewing the Coaches’ standardized weekly reports, holding weekly phone meetings, regular site visits, and communication with school principals and the OKDHS supervisor. Members of the UF team (KK, BL) and the CN team (MB, BG) participate in the calls twice a month and conduct regular site visits. Coaches were provided an initial 3-day training and two yearly booster trainings (scheduled at the beginning of fall and spring semesters). Training and feedback include guidance in implementation of SBIRT, motivational interviewing techniques, and cultural sensitivity training with special emphasis on motivational interviewing with Native Americans.

To support and expand reach of the SBIRT intervention, additional school and community members involved with youth are provided *Connector* training to enhance their connections and communication with youth, identify signs of high risk, and refer youth to school and community resources, if needed. This aspect of the intervention serves to enhance school personnel and community member competency in communicating with adolescents about alcohol and other important decisions, which aids in sustainability of any positive effects. Postcards with behavioral tips (Komro et al. 2008; Perry et al. 2003, Perry et al. 2002) are mailed quarterly to high school students’ primary residence, as well as posters distributed throughout the community and placed in commonly frequented venues such as restaurants, grocery stores and churches. Topics of the campaign are selected from scientifically established risk and protective factors for adolescent alcohol use and include (a) communication and connection, (b) monitoring, (c) identification of high-risk behaviors and community resources, (d) risks associated with alcohol use among youth, and (e) family rules.

## Research Design

### Study Design

Implementation and evaluation of complex community-based interventions requires careful selection of multiple design elements to optimize causal inference. We have designed this study to rigorously evaluate effects of the community-wide intervention on the primary ultimate outcomes of alcohol use, misuse and alcohol-related problems, while also remaining within strict limitations on study resources. In addition to an overall estimate of intervention effect, the trial must evaluate effects of each major intervention strategy (here CMCA and CONNECT) on intermediate (or proximal) outcomes. As one example, the CONNECT intervention will target alcohol expectancies and social support, while the environmental intervention will target commercial and social access to alcohol. To achieve even these proximal outcomes, it is necessary to measure whether interventions are implemented with fidelity and the extent to which target audiences are being effectively reached. To address these questions we also include in the study design a rigorous assessment of intervention implementation.

Six key design elements optimize causal inference and experimental evaluation of the intervention (Kratochwill and Levin 2010; Shadish et al. 2002). As shown in Table 1, we have combined a controlled interrupted time-series design, purposive selection of towns, random assignment to study condition, nested cohorts as well as repeated cross-sectional observations, a factorial design crossing two conceptually distinct interventions, and multiple comparison groups.

**Table 1:** Randomized time-series experimental design for the Prevention Trial in the Cherokee Nation

A. Treatment Town: (*n* ≈ 250 youth)	O(M)_ikt1(1)_………O(M)_ikt5(12)_	X^CONNECT^_6(13)–19(54)_	O(M)_ikt6(13)_………………..…_19(54)_
B. Treatment Town: (*n* ≈ 250 youth)	O(M)_ikt1(1)_………O(M)_ikt5(12)_	X^CMCA^_6(13)–19(54)_	O(M)_ikt6(13)_…………..………_19(54)_
C. Treatment Towns (*n* = 2): (*n* ≈ 500 youth)	O(M)_ikt1(1)_………O(M)_ikt5(12)_	X^COMBINED^_6(13)–19(54)_	O(M)_ikt6(13)_……….....………._19(54)_
D. Control Towns (*n* = 2): (*n* ≈ 500 youth)	O(M)_ikt1(1)_……………………………..……..…………………….….……..………….O(M)_ikt19(54)_		
E. Control Towns (*n* = 6): (*n* ≈ 250 youth/town)	O(A)_ikt1(1)_………………………..……..…………………….….…………………..…..…O(A)_ikt5(54)_		

*i* individual/person, *k* treatment condition, *t* time, *O* observation, *O*(*M*) monitoring system and archival sources, quarterly (monthly) observations/measures + archival sources, *O*(*A*) archival sources of yearly (monthly) observations/measures

The large number of repeated measures (a time-series) substantially increases internal validity (i.e., strength of causal inference) as well as statistical power over conventional pre/post community trial designs. One of our previous community trials clearly established the feasibility and high level of inferential utility of such time-series designs (Wagenaar et al. 2005). The study sample will include a cohort of high-school students within study towns (class of 2015 and 2016). Quarterly measurements of students via a school-based survey and monthly assessments of alcohol purchase attempts across all study treatment and control sites produce a time-series design with observations of youth and alcohol outlets nested within town over a 4-year period (there is one high school per town). In addition to repeated cross-sectional samples of youth, we will use participant identifiers to track the embedded cohorts over 4 years. Quarterly and monthly measurements will provide powerful tools to examine functional relations between the introduction and ramp-up of interventions and relevant outcomes over time.

Towns were purposively selected and randomly assigned to study condition. Of the rural towns within the 14-county Cherokee Nation tribal jurisdiction area that have high schools, 12 met the study selection criteria, which included: (a) served by one mid-sized high school with 400–700 students (which means that the high school student population is socially integrated into the town); (b) at least a 30-mile separation from the next town (to limit the threat of contamination across study conditions; these are socially cohesive communities where people tend to stay within a 20-mile radius of their town and identify strongly with the community); and (c) have local businesses, including ones that sell alcohol (so that local initiatives could target commercial sources of alcohol). We constructed a risk score for each town based on school and town characteristics obtained from school and census records, as well as the Youth Risk Behavior Survey, and selected four of the six among the highest risk communities. These four towns were then randomly assigned into one of the four study conditions (A–D in Table 1). After the initial three baseline youth surveys were implemented within the first 9 months of the project, we updated power calculations with actual data and decided to recruit two additional towns, allocating one to the combined intervention condition and one to the control condition to ensure adequate youth sample size for cohort analyses. These were drawn from the initial list of 12 eligible towns and selected based on proximity to the original combined and control towns. Therefore, the final sample includes six towns, two each in the combined and control conditions and one each in the CMCA only and CONNECT only conditions. The remaining 6 towns will serve as archival controls (condition E in Table 1). Study procedures were approved by both the University of Florida and Cherokee Nation Institutional Review Boards.

### Recruitment

Towns, with their embedded high school, were randomly assigned to study condition prior to recruitment. Following randomization to condition, we (UF and CN team leaders) scheduled recruitment meetings with school district superintendents and high school principals. Information packets specific to each condition were presented to the school leadership, as well as a summary of the entire project, and a cooperative agreement form for them to sign. All six schools agreed to participate in the study and signed cooperative agreement forms to participate in the study for 4 years. Response rates at the individual level (for the five baseline waves of student surveys) ranged from 82 % to 87 %.

### Study Communities, Schools, Participants

The six study towns range in population from 1,423 to 9,300, with 9 % to 37 % of the town population being Native American. Median household income ranges from $26,222 to $38,000, below median income levels for Oklahoma ($44,287) and the U.S. ($52,762).

On the study survey, students are able to mark all that apply for race/ethnicity. Forty-seven percent of cohort students (school range 40 % to 63 %) indicated they were Native American, including 23 % who reported being Native American only, 21 % Native American and White, and 3 % Native American and another race/ethnicity. Seventy-one percent of the students reported being White (school range 53 % to 78 %), including 45 % who reported being White only. Fifty-three percent of students (school range 47 % to 66 %) reported being eligible for free/reduced price lunch, and the mean age was 15 overall and for each school.

### Data Collection

#### Youth Survey

Brief (10–15 min) self-report questionnaires are administered to the study cohort (9th and 10th grade students during the 2012–13 academic year, class of 2015 and 2016) in the six study schools four times each year (approximately in October, December, February, May). The questionnaires are administered under the direction of the study Evaluation Director (TK) with a team of locally hired (not from the study communities) and trained research survey staff following standardized procedures. In order to maximize student confidentiality, school staff are not involved in survey implementation. Students receive a $5 incentive for each survey, and an additional $10 during the fourth administration if they participated in all surveys for which they were eligible during the academic year. Each questionnaire has a unique study ID to link individual responses over time. Parents are sent a consent letter 4–6 weeks prior to survey administration and asked to call a toll-free number or return a postage paid postcard if they do not want their child to participate. Prior to the consent letter mailing, we send a postcard informing parents that an important letter is being mailed to them, and then following the consent mailing, we send a reminder postcard. Students are given an assent form and given the opportunity to refuse participation.

Questionnaire items are based on the national Youth Risk Behavior Survey (YRBS) (Centers for Disease Control and Prevention 2010), the Oklahoma Prevention Needs Assessment Survey (OPNA; Oklahoma Department of Mental Health and Substance Abuse Services 2010), and surveys used in the Project Northland trials (Komro et al. 2008; Perry et al. 1996). The main outcome of interest is alcohol use, measured primarily with three standard items from the YRBS, including frequency of use in one’s lifetime, past 30 days, and 5 or more drinks of alcohol in a row in the past 30 days. We also included items on smokeless tobacco, cigarettes, marijuana, prescription drugs without a doctor’s prescription, and any other illegal drug (Centers for Disease Control and Prevention 2010).

In Oklahoma high schools, the state department of mental health and substance abuse services implements the Oklahoma Prevention Needs Assessment Survey every even-numbered year and the Youth Risk Behavior Survey every odd-numbered year. These annual surveys implemented in the archival control towns will be used as an additional secondary comparison group. Comparisons will also be made with state and national trends.

Among 9th and 10th grade students during baseline wave 5 (December 2012; *n* = 1,562), use in the past month was reported by 19 % for alcohol use, 12 % for binge drinking, 10 % for chewing tobacco use, 15 % for cigarette use, and 9 % for marijuana use. Alcohol and marijuana use rates among 9th and 10th graders in the six project schools were slightly lower than rates reported from the 2011 Oklahoma and U.S. Youth Risk Behavior Survey.

#### Alcohol Purchase Attempts

The propensity of stores to sell alcohol to underage youth is measured directly using a protocol well-developed in our previous trials (Komro et al. 2008; Perry et al. 1996; Wagenaar and Perry 1994; Wagenaar et al. 2005), a protocol that is now in widespread use nationally. Buyers are 21 or older but appear younger than 21 and are trained to request a standard type of alcohol without identification as part of the uniform protocols in making the purchase attempt. Off-site alcohol outlets in each of the study communities are assessed once each month. The mean number of alcohol outlets per town is 21 (range 17 to 25). (Outlets were included if they were within a 20-min drive time from the town center.) Buyers are accompanied by another young-appearing research staff member, both complete data collection forms following each purchase attempt, and they are debriefed about their experiences using standardized forms. The procedure directly assesses how strictly proof-of-age is adhered to in the intervention and control communities. Baseline buy rate ranged from 17 % to 32 %.

#### Archival Data

In all 12 study towns, we collect archival data (e.g., traffic citations, police arrest reports, juvenile justice system reports, alcohol-related fatal and non-fatal crashes, teen STIs, teen birth rate, juvenile substance abuse treatment admissions), including high school records and annual surveys (e.g., absenteeism, suspensions, juvenile offenders, senior graduation rate, 4-year drop out rate, standardized test scores, annual other alcohol and drug survey data). These data provide a baseline characterization of the study and comparison towns and enable monitoring of outcomes during the study and after intervention implementation.

#### Implementation Evaluation

Building on our prior work, we developed a standardized system to collect implementation measures daily as a routine part of the field intervention staff activities. A user-friendly measurement system is not only vital to an overall evaluation effort; it also functions as a management information system for field intervention staff and as a continuous quality improvement system to improve the implementation and effectiveness of interventions over time. Field intervention staff electronically access a secure, password protected encrypted reporting system modeled on similar systems developed for our previous trials, but enhanced with the use of the latest in current easy to use database software (i.e., Bento) (Komro et al. 2008; Perry et al. 2000a; Wagenaar et al. 2000a, b; Wagenaar and Perry 1994). In addition to using the system as an integral tool to facilitate their daily work tasks, the staff perform a more focused weekly update followed by securely transferring the week’s updated data to the research team.

## Discussion

This trial contributes to the field of prevention and translation science with innovative design features, a rigorous evaluation of a population-level implementation of SBIRT supplemented with family and community components, and a replication of an efficacious environmental change approach within high-risk, underserved communities with large populations of Native Americans. The project will provide guidance on the effectiveness of intervention components alone and in combination, and advance the understanding of effective strategies for underage drinking prevention among Native American youth, as well as high-risk rural youth in general.

The trial also provides a successful example of a true academic–community partnership in the design and implementation of a rigorous community randomized trial funded by NIH. Our CBPR team reflects a close and effective collaboration between scientists at the University of Florida, with a long history of conducting community intervention trials, and the leadership of Cherokee Nation Behavioral Health, with strong interests in promoting youth health and advancing preventive intervention effectiveness. We, the CN and UF team, are conducting an NIH-funded community trial with detailed attention to rigorous scientific methods, at the same time developing strong connections with local community-based organizations. We are accessible, communicative, and responsive to questions, priorities and concerns of our community-based partners. We are in frequent contact with the OKDHS and school principals across the Cherokee Nation as we implement this complex intervention trial. Each year we also hold more formal meetings with school leadership to share school-level data from the student surveys and provide updates on intervention progress. We support our community action teams with frequent communications, strategic planning, review of intervention materials, and response to requests for information regarding effective strategies. The action teams continue to build additional partnerships with local citizens and organizations to initiate and sustain community change. The CONNECT Coaches receive intensive training in MI, a culturally sensitive approach to communicating with and positively influencing youth, with intended diffusion to school, family and community members. The CBPR framework and novel approaches to implementation, we hope, will contribute to long-term positive outcomes for the youth in the study and long-term sustainability to continue these efforts when NIH funding ends.

Developing strong partnerships between prevention scientists and community prevention leaders, as well as technical skill, knowledge and leadership development across multiple community-based organizations provides a foundation for sustainability of effective prevention strategies, as well as continued research initiatives. We have created a positive example of an effective CBPR approach that is combined with a rigorously implemented experimental trial.

Besides a generalizable community-university partnership, our community prevention leaders work for and are members of a Native American tribe. Historical cultural losses, discrimination, economic deprivation, and the accumulation of stressful life events have led to multiple and significant health disparities among Native Americans and underscore the need for culturally sensitive and responsive approaches to address these disparities. It is our hope that our tribal-university research partnership provides a template for other such partnerships to tackle difficult health disparity issues with relevant and responsive community action research. We agree with and actively support the call for additional tribal-university partnerships that “focus on making a difference in the lives of Indigenous people” (Whitbeck et al. 2014, p. 214), and for creating and embracing equal partnerships.
